# Position/Attitude Cascade Regulation of Nonholonomic Four-Wheeled Mobile Robot via Double-Loop Sliding-Mode Control Mechanism

**DOI:** 10.3390/e25010027

**Published:** 2022-12-23

**Authors:** Xin Zhang, Dongchen Qin, Shuting Wang, Yuanlong Xie, Hu Li, Shiqi Li

**Affiliations:** 1 School of Mechanical and Power Engineering, Zhengzhou University, Zhengzhou 450001, China; 2 School of Mechanical Science and Engineering, Huazhong University of Science and Technology, Wuhan 430074, China; 3 Hubei Key Laboratory of Advanced Control and Intelligent Automation for Complex Systems, Wuhan 430074, China; 4 Engineering Research Center of Intelligent Technology for Geo-Exploration, Ministry of Education, Wuhan 430074, China

**Keywords:** nonholonomic four-wheeled mobile robot, double-loop sliding-mode control, nonsingular terminal sliding-mode surface, barrier function

## Abstract

Nonholonomic four-wheeled mobile robot (NFMR) is a typical multiple input–multiple output system that formulates its kinematic dynamics concerning position and attitude in a parallel manner. However, due to the lumped disturbances and interconnected states, demand-satisfied performance is difficult to obtain for existing coupled control solutions. To address this problem, a double-loop sliding-mode control (DLSMC) mechanism is proposed for achieving position/attitude cascade regulation. For the outer position tracking loop in the proposed scheme, a sliding mode control method of the bounded time-varying integral nonsingular terminal is designed to guarantee fast tracking in the presence of large initial errors and input saturation. On the other hand, for the inner attitude control loop, a novel adaptive barrier function-based sliding-mode control method is proposed without control gain overestimation. This enables the attitude to follow within a predefined vicinity of the sliding mode surface and holds it subsequently independent of the lumped uncertainties. Theoretical analysis is conducted to demonstrate the asymptotic stability. Comparative experiments implemented on a homemade NFMR show enhanced trajectory tracking performance and system robustness using position/attitude cascade regulation via the proposed DLSMC mechanism.

## 1. Introduction

Due to its advantages in navigation safety, working consistency, and productivity, wheeled mobile robot (WMR) has become a key means of material transportation in modern factories. Among existing WMRs, the nonholonomic four-wheeled mobile robot (NFMR) achieves higher flexibility, mobility, and maneuverability when navigating narrow environments or confined spaces [1]. The nonholonomic property and system uncertainties of NFMR impose great challenges for accurate trajectory tracking in hash industrial environments [2]. In addition, non-negligible initial errors in terms of position and attitude tracking may mitigate the control performance when the original state of the concerned NFMR is far from the planned trajectory. In practice, precise and robust tracking control needs to consider position and attitude control errors simultaneously. Accordingly, this study aims to design a control mechanism for the synergetic convergence of position and attitude regulation when the concerned NFMR is subjected to large initial errors and unknown uncertainties.

The controller design of WMRs has attracted considerable research attention. The NFMR contains kinematic and dynamic models [3]. Designing a dynamic controller can be difficult because of the complexity of the dynamics and the difference between the wide range of mobile robots [4]. The NFMR may contain different dynamic models for a distinctive mechanical structure. On the other hand, the kinematic equations are the same to ensure compatibility with multimodal features [5]. This implies that the tracking control of the NFMR can be simple and easy to implement when kinematic model-based solutions are employed [6,7]. Currently, typical tracking control methods include proportional integral control, model predictive control [8,9], adaptive control [10], and sliding mode control (SMC) [11]. Among these, the SMC method is able to achieve better control robustness against uncertainties and disturbances [12,13,14]. In practice, SMC offers potential applicability in simultaneous regulations of position and attitude of the NFMR because of its easy implementation and system robustness. For instance, a coupled SMC solution with three virtual control gains was proposed for lateral and longitudinal stabilization [15]. However, the longitudinal error will be interrupted when the lateral error converges to zero. A coupled sliding mode surface was constructed with longitudinal and attitude errors to track the desired trajectory of the NFMR [16]. To conclude, new coupled problems introduced by the above method are inapplicable in the case of large initial errors. Moreover, these methods are sensitive to tracking curvature changes, making it difficult to guarantee synchronous coordination between the convergence of lateral and longitudinal errors. 

On the other hand, among the existing SMC-based investigations used in WMRs, linear and terminal sliding mode surfaces have been widely adopted [17,18]. For one thing, to improve existing first-order SMC methods [19,20,21] for the NFMR, the integral SMC method was introduced into the NFMR controller design [22,23,24]. The initial error was further taken into account in the integral SMC design to enhance the achievement of convergence [25,26,27]. Furthermore, the combination of integral and terminal sliding modes not only solves the singularity problem of the terminal sliding mode but also ensures finite time convergence [25]. However, input saturation of the NFMR is ignored in the SMC studied above. In practice, mandatory input upper bound constraints are widely used. This may affect the smoothness of the control input sequence, thereby having a negative effect on the actuation performance or even damaging the actuator systems. In addition, to eliminate chattering caused by system uncertainties and unknown disturbances, different resolution methods have been proposed in SMC, such as ESO [13,28,29,30,31], disturbance modeling [12], and adaptive sliding mode control (ASMC) [14,32,33,34]. However, in these methods, it is necessary to obtain the prior information of disturbance supremum, which is not easy to obtain for a real-time application.

Consequently, we propose a double-loop control solution to provide the developed NFMR with outstanding trajectory-tracking performance and system robustness. The contributions of this study are presented as follows: (1) Compared with single-loop control methods [15,16], a novel DLSMC mechanism is presented to realize the position/attitude cascade regulation. The proposed mechanism solves not only the nonholonomic problem but also achieves different control requirements for positional and attitude tracking; (2) A modified adaptive barrier function SMC (ABFSMC) with gains regulated by barrier function for the inner control loop is proposed to suppress lumped disturbances without the a priori upper information. The derived control law presents better anti-chattering performance than the linear or terminal reaching law while guaranteeing the robustness of the whole control loop; (3) An enhanced bounded time-varying integral nonsingular terminal SMC (BTINTSMC) method with a time-varying sliding mode surface for the outer control loop that considers both input saturation and finite time convergence is presented to ensure the continuity of the input signal. This method demonstrates better convergence than the nonsingular terminal SMC method [21] and the constant rate SMC method [19]; (4) Comparative results verified that the proposed mechanism outperforms existing ones in terms of initial error convergence and trajectory tracking accuracy of NFMR control under the condition of restrained input. 

The remainder of this paper is structured as follows. The kinematic model of the considered NFMR and problem formulation are discussed in Section 2. The proposed DLSMC mechanism with theoretical analysis is presented in Section 3, including the designed BTINTSMC for the outer loop and ABFSMC for the inner loop. Experimental verifications are demonstrated in Section 4. Finally, the conclusions of this study and future investigations are provided in Section 5.

## 2. Kinematic Modelling and Problem Statement

### 2.1. Kinematic Modelling of the NFMR

**Figure 1:**
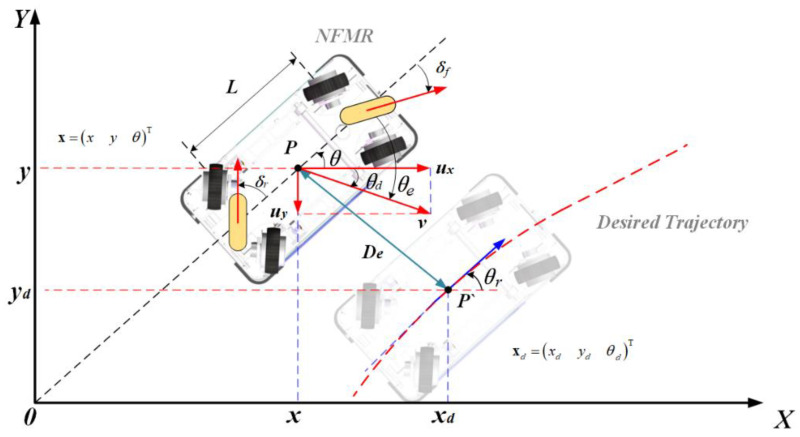
Model of the NFMR with position/attitude cascade regulation mechanism.

As shown in Figure 1, there is the chassis plane figure of realistic NFMR under study. Its motion can be simplified into a virtual single-car model whose two virtual wheels are located on the center line of the robot body [1]. The steering angles of the front and rear wheels are denoted by $\delta_{f}$ and $\delta_{r}$. The point P is the geometric center of the robot body, so its position and attitude can be denoted by (x, y) and θ, respectively. Point P’ is the target point on the desired trajectory, which gives the desired position $\left(x_{d},y_{d}\right)$. $\theta_{r}$ is the angle of tangent direction of point P’. $u_{x}$ and $u_{y}$ represent the proper virtual linear velocity control inputs of NFMR in the X and Y direction to track the point P’, respectively, which are given by the controller. Then the realistic linear velocity input v is given by $v=\sqrt{u_{x}^{2}+u_{y}^{2}}$. $\theta_{d}$ is the direction of v and is given by $\theta_{d}=\mathrm{atan}2(u_{y}/u_{x})$ with $u_{x}\neq 0$. In Figure 1, the given direction of $u_{y}$ and $\theta_{d}$ are the negative axes. Both $\theta_{d}$ and $\theta_{r}$ are selected as the desired attitude. $\theta_{d}$ is regarded as the direct desired attitude, which is tracked via the controller, while $\theta_{r}$ is seen as the indirect one, which is tracked via self-adjustment of mobile robot attitude during trajectory tracking. For excellent tracking performance, the attitude of NFMR should not only track $\theta_{d}$ accurately but also have good tracking results for $\theta_{r}$. As defined above, the NFMR system state is $x={\left[x,\;y,\;\theta \right]}^{T}$, then kinematic states of NFMR can be expressed as

(1) $$\dot{x}=\left[\begin{array}{c} \dot{x}\\ \dot{y}\\ \dot{\theta } \end{array}\right]=\left[\begin{array}{cc} \cos\theta & 0\\ \sin\theta & 0\\ 0 & 1 \end{array}\right]\left[\begin{array}{c} v\\ \frac{\left(j_{1}+1\right)v\tan\delta_{f}}{j_{1}L+j_{2}L} \end{array}\right],$$

where L denotes the robot length, $j_{1}$ and $j_{2}$ denote the user-defined configuration parameters, $j_{1}=\tan(\delta_{r})/\tan(-\delta_{f})$. If $j_{1}=0$ and $j_{2}=1$, then only the front wheels are turned for steering, that is, the NFMR is operated in the conventional Ackerman mode. Meanwhile, if $j_{1}=1$ and $j_{2}=0$, then the steering angle of the front and rear wheels are opposite, implying that the double-Ackerman steer mode is applied. This mode can reduce the steering radius and obtain a fast yaw response in narrow spaces.

Let

(2) $$\omega =\frac{\left(j_{1}+1\right)v\tan\delta_{f}}{\left(j_{1}L+j_{2}L\right)}.$$

The nonholonomic system (1) can be restated as

(3) $$\{\begin{array}{l} \dot{x}=v\cos\theta \\ \dot{y}=v\sin\theta \\ \dot{\theta }=\omega \end{array}.$$

The desired trajectory $\left(x_{d},y_{d}\right)$ is determined by

(4) $$x_{d}\left(t\right)=f\left(t\right),$$

(5) $$y_{d}\left(t\right)=g\left(x_{d}\left(t\right)\right),$$

where f(⋅) and g(⋅) are sufficiently smooth functions with f(⋅)≥0 and $\dot{f}(\cdot )>0$, respectively. Then $v_{d}$ and $\theta_{r}$ can be given by

(6) $$v_{d}=\sqrt{{\dot{x}}_{d}^{2}+{\dot{y}}_{d}^{2}},$$

(7) $$\theta_{r}=\mathrm{atan}2\left(\frac{{\dot{y}}_{d}}{{\dot{x}}_{d}}\right).$$

To avoid interconnected kinematic states, in this paper, the system state error $x_{e}$ is described in the global frame and selected $\theta_{d}$ as the desired attitude [19].

Only when the NFMR moves with $\theta =\theta_{d}$, can the nonholonomic system track the target point under v. Therefore, the system error state $x_{e}$ is then calculated using

(8) $$x_{e}=\left[\begin{array}{c} x_{e}\\ y_{e}\\ \theta_{e} \end{array}\right]=\left[\begin{array}{c} x-x_{d}\\ y-y_{d}\\ \theta -\theta_{d} \end{array}\right].$$

Accordingly, a decoupled dynamics is obtained as follows

(9) $$\begin{array}{l} {\dot{x}}_{e}=\dot{x}-{\dot{x}}_{d}=u_{x}-v_{d}\cos\theta_{r}\\ {\dot{y}}_{e}=\dot{y}-{\dot{y}}_{d}=u_{y}-v_{d}\sin\theta_{r}\\ {\dot{\theta }}_{e}=\dot{\theta }-{\dot{\theta }}_{d}=\omega -{\dot{\theta }}_{d}+\varpi \end{array}$$

where ϖ denotes the lumped disturbance consisting of modeling uncertainties and external disturbances. In practice, ϖ is assumed to be bounded reasonably, such that $\left|\varpi \right|<\eta_{1}$, where $\eta_{1}\in {\mathbb{R}}^{+}$ denotes a positive constant.

**Remark 1.**  *In the existing research,*$\theta_{r}$*is selected as the desired attitude instead of*$\theta_{d}$*, and the system state error*$x_{e}$*is often described in the local frame* [16]*. The resulting error dynamics are interconnected.*

### 2.2. Problem Statement

The primary control challenges of the NFMR system in achieving precise trajectory tracking are presented as follows: (1) interconnected kinematic states of NFMR in terms of position and attitude, (2) suitable control law to guarantee the stability of initial error convergence, and (3) input saturation issue. Existing solutions for trajectory tracking of the NFMR typically use the coupled single-loop control mechanism. However, the single-loop control mechanism usually performs unsatisfactorily in the initial tracking stage because the precise and simultaneous control of the convergence of position and attitude tracking is difficult. 

Besides, input signals u, where $u={\left(v,\;\omega \right)}^{T}$, are restricted to some regions of action, that is, $0<v_{\min}<v<v_{\max}$ and $\left|\omega \right|<\omega_{\max}$, for practicality. The derivation of the control law allows the system error $x_{e}$ to converge to zero. This study aims to design a DLSMC mechanism with bounded input and fast convergence of initial error, such that the system error $x_{e}$ is Lyapunov-stable and distance-optimal when returning to the desired trajectory from the initial state. Thus, the novel control input established with v and ω can successfully handle the tracking problems mentioned above. We propose a DLSMC mechanism in this study to achieve a position/attitude cascade control framework and precise trajectory tracking performance.

## 3. Main Results

### 3.1. Overall Control Mechanism

**Figure 2:**
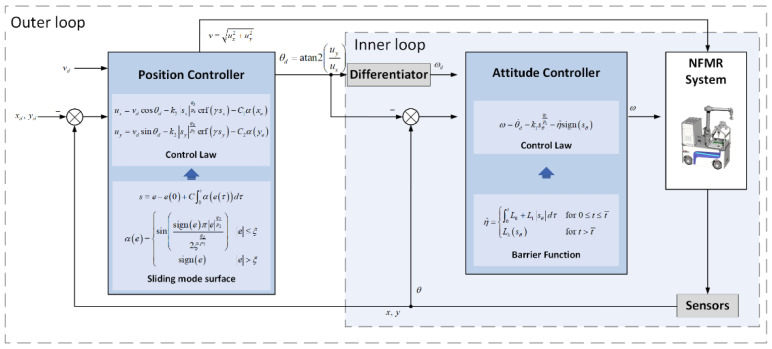
Overall control architecture.

The proposed DLSMC mechanism (Figure 2) consists of (1) a position controller of the outer loop with a BTINTSMC method that serves as the tracking control of the position tracking subsystem proposed for solving the problem of input saturation, and (2) an attitude controller of the inner loop with the ABFSMC method to ensure the steady and precise tracking of the desired attitude. Global convergence of the DLSMC mechanism is proved by constructing the Lyapunov function.

### 3.2. Attitude Controller of the Inner Loop

A new-type ABFSMC framework is designed in this section to allow the system state to converge into and thereafter stay within a predefined range near the original point. Considering the orientation kinematics determined by (9), let the sliding mode function be defined as

(10) $$s_{\theta }=\theta_{e}.$$

The following ABFSMC law is constructed

(11) $$\omega ={\dot{\theta }}_{d}-k_{1}s_{\theta }^{\frac{q_{1}}{p_{1}}}-\hat{\eta }\mathrm{sign}\left(s_{\theta }\right),$$

where $k_{1}$ is a positive constant coefficient, $\hat{\eta }$ is the adaptive error compensation to be designed later, $p_{1}$ and $q_{1}$ are positive odd numbers, and $1<p_{1}/q_{1}<2$.

**Figure 3:**
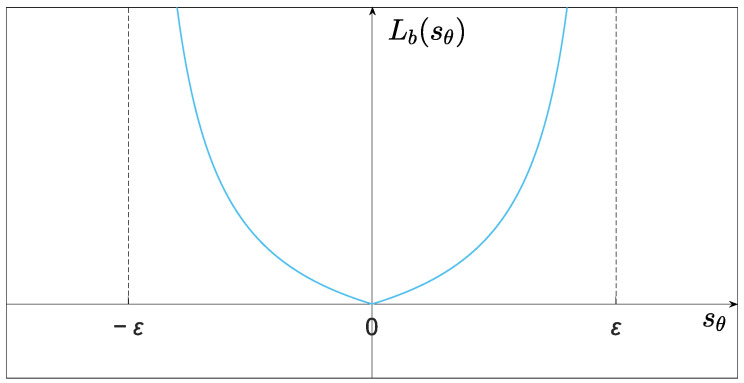
Barrier function $L_{b}\left(s_{\theta }\right)$ for $s_{\theta }\in \left(-\varepsilon ,\;\varepsilon \right)$.

With a small positive angle ε whose size is defined by equalizing the anti-disturbance performance and operation speed. Define $\bar{t}$ as the first time for $s_{\theta }$ reaching the region [−ε/2, ε/2] from any initial value. If $\left|s_{\theta }(0)\right|\leq \varepsilon /2$, then $\bar{t}=0$. The error compensation $\hat{\eta }$ of the ABFSMC law is then expressed as

(12) $$\hat{\eta }(t)=\{\begin{array}{ll} \int_{0}^{t}(L_{0}+L_{1}\left|s_{\theta }\right|)d\tau & \mathrm{for}\;0\leq t\leq \bar{t}\\ L_{b}\left(s_{\theta }\right) & \mathrm{for}\;t>\bar{t} \end{array},$$

where $L_{0}>0$ and $L_{1}>1$ denote the control parameters, $L_{b}\left(s_{\theta }\right)$ is the BF (Figure 3) designed as

(13) $$L_{b}\left(s_{\theta }\right)=\begin{array}{cc} \frac{\lambda \left|s_{\theta }\right|}{\varepsilon -\left|s_{\theta }\right|} & ,\;s_{\theta }\in \left(-\varepsilon ,\;\varepsilon \right) \end{array},\;\lambda >0,$$

where λ is a positive constant coefficient.

The adaptive error compensation $\hat{\eta }$ is upper limited by $\hat{\eta }\leq \eta_{2}$, where $\eta_{2}\in {\mathbb{R}}^{+}$ denotes a positive constant. Let $\eta =\max\{\eta_{1},\;\eta_{2}\}$ (explain in Remark 2). We provide the following theorem.

**Theorem 1.**  *For the formulated NFMR system (9), under the proposed control law (11) with adaptive error compensation defined by (12) and (13), the tracking error of the inner control loop can converge into a small neighborhood of the equilibrium point, namely,*$\theta_{e}\in \left[-\varepsilon ,\;\varepsilon \right]$.

**Proof.**  First, the derivative of the sliding mode variable $s_{\theta }$ is computed as

(14) $$\begin{array}{cc} {\dot{s}}_{\theta } & ={\dot{\theta }}_{e}=\omega -{\dot{\theta }}_{d}+\varpi \\ & ={\dot{\theta }}_{d}-k_{1}s_{\theta }^{{q_{1}/p}_{1}}-\hat{\eta }\mathrm{sign}\left(s_{\theta }\right)-{\dot{\theta }}_{d}+\varpi \\ & =-k_{1}s_{\theta }^{{q_{1}/p}_{1}}-\hat{\eta }\mathrm{sign}\left(s_{\theta }\right)+\varpi \end{array}$$

Since two situations $\left|s_{\theta }\right|\geq \varepsilon /2$ and $\left|s_{\theta }\right|<\varepsilon /2$ can be regarded as two respective situations of the adaptive law in (12) and (13), the convergence of the $\theta_{e}$ subsystem is considered as the following two cases.

Case (1) $0\leq t\leq \bar{t}$

According to (12), the Lyapunov function $V_{1}$ is constructed

(15) $$V_{1}=\frac{1}{2}s_{\theta }^{2}+\frac{1}{2}{\left(\hat{\eta }-\eta \right)}^{2}.$$

Based on (14) and the proposed ABFSMC law, we have

(16) $$\begin{array}{lll} {\dot{V}}_{1} & = & s_{\theta }{\dot{s}}_{\theta }+\left(\hat{\eta }-\eta \right)\dot{\hat{\eta }}\\ & = & s_{\theta }\left(-k_{1}\mathrm{sign}\left(s_{\theta }\right){\left|s_{\theta }\right|}^{{q_{1}/p}_{1}}-\hat{\eta }\mathrm{sign}\left(s_{\theta }\right)+\varpi \right)+\left(\hat{\eta }-\eta \right)\left(L_{0}+L_{1}\left|s_{\theta }\right|\right)\\ & = & -k_{1}{\left|s_{\theta }\right|}^{{q_{1}/p}_{1}+1}-\hat{\eta }\left|s_{\theta }\right|+\varpi s_{\theta }+L_{1}\left(\hat{\eta }-\eta \right)\left|s_{\theta }\right|+L_{0}\left(\hat{\eta }-\eta \right)\\ & \leq & -k_{1}{\left|s_{\theta }\right|}^{{q_{1}/p}_{1}+1}-\hat{\eta }\left|s_{\theta }\right|+\left|\varpi \right|\left|s_{\theta }\right|+L_{1}\left(\hat{\eta }-\eta \right)\left|s_{\theta }\right|+L_{0}\left(\hat{\eta }-\eta \right)\\ & \leq & -k_{1}{\left|s_{\theta }\right|}^{{q_{1}/p}_{1}+1}-\hat{\eta }\left|s_{\theta }\right|+\eta \left|s_{\theta }\right|+L_{1}\left(\hat{\eta }-\eta \right)\left|s_{\theta }\right|+L_{0}\left(\hat{\eta }-\eta \right)\\ & = & -k_{1}{\left|s_{\theta }\right|}^{{q_{1}/p}_{1}+1}-\left(\left(1-L_{1}\right)\left|s_{\theta }\right|-L_{0}\right)\left(\hat{\eta }-\eta \right) \end{array}.$$

According to (12), one can conclude that $\hat{\eta }\leq \eta$, $L_{0}>0$ and $L_{1}>1$. If we choose $\hat{\eta }-\eta =-\left|\hat{\eta }-\eta \right|$ and $1-L_{1}=-\left|1-L_{1}\right|$, we obtain

(17) $${\dot{V}}_{1}\leq -k_{1}{\left|s_{\theta }\right|}^{{q_{1}/p}_{1}+1}-\left(\left|1-L_{1}\right|\left|s_{\theta }\right|+L_{0}\right)\left|\hat{\eta }-\eta \right|.$$

Since A>0 and B>0 with $A=\sqrt{2}k_{1}{\left|s_{\theta }\right|}^{{q_{1}/p}_{1}}$ and $B=\sqrt{2}\left(\left|1-L_{1}\right|\left|s_{\theta }\right|+L_{0}\right)$ when $s_{\theta }\neq 0$, the following equation can be obtained

(18) $$\begin{array}{lll} {\dot{V}}_{1} & \leq & -A\frac{\left|s_{\theta }\right|}{\sqrt{2}}-B\frac{\left|\hat{\eta }-\eta \right|}{\sqrt{2}}\\ & \leq & -\chi_{1}\left(\frac{\left|s_{\theta }\right|}{\sqrt{2}}+\frac{\left|\hat{\eta }-\eta \right|}{\sqrt{2}}\right)\\ & \leq & -\chi_{1}V_{1}^{\frac{1}{2}} \end{array},$$

where

(19) $$\chi_{1}=\min\left\{A,\;B\right\}.$$

For $\chi_{1}>0$, a positive constant $\Omega_{1}$ will satisfy the following inequality

(20) $$\Omega_{1}\leq \chi_{1}\left(t\right),\forall \left|s_{\theta }\left(t\right)\right|>\frac{\varepsilon }{2}.$$

The integration of (18) and (20) leads to

(21) $${\dot{V}}_{1}\leq -\Omega_{1}V_{1}^{\frac{1}{2}},$$

which then obtains

(22) $$\bar{t}\leq \frac{V_{1}^{\frac{1}{2}}\left(0\right)-V_{1}^{\frac{1}{2}}\left(\bar{t}\right)}{0.5\Omega_{1}}.$$

Finite-time convergence is clearly guaranteed by (22), thereby indicating that $s_{\theta }\left(t\right)$ reaches the region of [−ε/2, ε/2] for the first time when $t=\bar{t}$. The adaptive gain will subsequently be transformed into the BF-based form for $t>\bar{t}$. Therefore, $s_{\theta }$ will converge to [−ε/2, ε/2] in the finite time $\bar{t}$ in this case under ABFSMC laws (11)–(13).

Case (2) $t>\bar{t}$

The following intermediate variable ψ is designed to illustrate the disturbance compensation effect using the error compensation $L_{b}\left(s_{\theta }\right)$. Of the presented ABFSMC law

(23) $$\psi =\varepsilon \frac{\left|\varpi \right|}{\left|\varpi \right|+\lambda }<\varepsilon .$$

It is noted that ψ is regulated by the time-varying disturbance ϖ and $L_{b}\left(\psi \right)=\left|\varpi \right|$. According to Case 1, we have $\left|s_{\theta }\left(\bar{t}\right)\right|=\varepsilon /2$. If $\left|s_{\theta }\left(\bar{t}\right)\right|=\varepsilon /2>\psi$, then the ABFSMC law allows $s_{\theta }\left(t\right)$ to satisfy $\left|s_{\theta }\left(t\right)\right|\leq \psi$ in a finite time period $t_{a}$ and ensures that $L_{b}\left(s_{\theta }(t)\right)>\left|\varpi \right|=L_{b}\left(\psi \right)$. The sliding mode variable $s_{\theta }$ will then meet $\left|s_{\theta }\left(t\right)\right|\leq \psi <\varepsilon$ for all $t>\bar{t}+t_{a}$. Meanwhile, if $\left|s_{\theta }\left(t\right)\right|=\varepsilon /2\leq \psi$, then $t_{a}=0$ and $\left|s_{\theta }\left(t\right)\right|\leq \psi <\varepsilon$ will exist for all $t>\bar{t}$.

Considering $\left|s_{\theta }\left(\bar{t}\right)\right|=\varepsilon /2>\psi$, a Lyapunov function $V_{2}$ is designed as

(24) $$V_{2}=\frac{1}{2}s_{\theta }^{2}+\frac{1}{2}L_{b}{\left(s_{\theta }\right)}^{2}.$$

The combination of (14) and the proposed ABFSMC law results in

(25) $$\begin{array}{cc} V_{2} & =s_{\theta }{\dot{s}}_{\theta }+L_{b}\left(s_{\theta }\right){\dot{L}}_{b}\left(s_{\theta }\right)\\ & =s_{\theta }\left(-k_{1}\mathrm{sign}\left(s\right){\left|s_{\theta }\right|}^{{q_{1}/p}_{1}}-L_{b}\left(s_{\theta }\right)\mathrm{sign}\left(s_{\theta }\right)+\varpi \right)\\ & +L_{b}\left(s_{\theta }\right)\frac{\lambda \varepsilon }{{\left(\varepsilon -\left|s_{\theta }\right|\right)}^{2}}\mathrm{sign}\left(s_{\theta }\right){\dot{s}}_{\theta }\\ & =-k_{1}{\left|s_{\theta }\right|}^{{q_{1}/p}_{1}+1}-L_{b}\left(s_{\theta }\right)\left|s_{\theta }\right|+\varpi s_{\theta }\\ & +L_{b}\left(s_{\theta }\right)\frac{\lambda \varepsilon }{{\left(\varepsilon -\left|s_{\theta }\right|\right)}^{2}}\mathrm{sign}\left(s_{\theta }\right)\left(-k_{1}\mathrm{sign}\left(s\right){\left|s_{\theta }\right|}^{{q_{1}/p}_{1}}-L_{b}\left(s_{\theta }\right)\mathrm{sign}\left(s_{\theta }\right)+\varpi \right)\\ & \leq -k_{1}{\left|s_{\theta }\right|}^{{q_{1}/p}_{1}+1}-L_{b}\left(s_{\theta }\right)\left|s_{\theta }\right|+\left|\varpi \right|\left|s_{\theta }\right|\\ & +L_{b}\left(s_{\theta }\right)\frac{\lambda \varepsilon }{{\left(\varepsilon -\left|s_{\theta }\right|\right)}^{2}}\left(-k_{1}{\left|s_{\theta }\right|}^{{q_{1}/p}_{1}}-L_{b}\left(s_{\theta }\right)+\varpi \mathrm{sign}\left(s_{\theta }\right)\right)\\ & \leq -k_{1}{\left|s_{\theta }\right|}^{{q_{1}/p}_{1}+1}-L_{b}\left(s_{\theta }\right)\left|s_{\theta }\right|+\left|\varpi \right|\left|s_{\theta }\right|\\ & -L_{b}\left(s_{\theta }\right)\frac{\lambda \varepsilon k_{1}{\left|s_{\theta }\right|}^{{q_{1}/p}_{1}}}{{\left(\varepsilon -\left|s_{\theta }\right|\right)}^{2}}-L_{b}\left(s_{\theta }\right)\frac{\lambda \varepsilon }{{\left(\varepsilon -\left|s_{\theta }\right|\right)}^{2}}\left(L_{b}\left(s_{\theta }\right)-\left|\varpi \right|\right)\\ & =-\left(k_{1}{\left|s_{\theta }\right|}^{{q_{1}/p}_{1}}+L_{b}\left(s_{\theta }\right)-\left|\varpi \right|\right)\left|s_{\theta }\right|\\ & -\left[\frac{\lambda \varepsilon k_{1}{\left|s_{\theta }\right|}^{{q_{1}/p}_{1}}}{{\left(\varepsilon -\left|s_{\theta }\right|\right)}^{2}}+\frac{\lambda \varepsilon }{{\left(\varepsilon -\left|s_{\theta }\right|\right)}^{2}}\left(L_{b}\left(s_{\theta }\right)-\left|\varpi \right|\right)\right]L_{b}\left(s_{\theta }\right) \end{array}$$

According to (13) and (23), $L_{b}\left(s_{\theta }\right)>L_{b}\left(\psi \right)=\left|\varpi \right|$ if $\left|s_{\theta }\left(t\right)\right|>\psi$. By defining

(26) $$\begin{array}{l} D=\sqrt{2}\left(k_{1}{\left|s_{\theta }\right|}^{{q_{1}/p}_{1}}+L_{b}\left(s_{\theta }\right)-\left|\varpi \right|\right)\\ E=\sqrt{2}\left[\frac{\lambda \varepsilon k_{1}{\left|s_{\theta }\right|}^{{q_{1}/p}_{1}}}{{\left(\varepsilon -\left|s_{\theta }\right|\right)}^{2}}+\frac{\lambda \varepsilon }{{\left(\varepsilon -\left|s_{\theta }\right|\right)}^{2}}\left(L_{b}\left(s_{\theta }\right)-\left|\varpi \right|\right)\right] \end{array}$$

one can obtain that

(27) $$\begin{array}{lll} {\dot{V}}_{2} & \leq & -D\frac{\left|s_{\theta }\right|}{\sqrt{2}}-E\frac{\left|L_{b}\left(s_{\theta }\right)\right|}{\sqrt{2}}\\ & \leq & -\chi_{2}\left(\frac{\left|s_{\theta }\right|}{\sqrt{2}}+\frac{\left|L_{b}\left(s_{\theta }\right)\right|}{\sqrt{2}}\right)\\ & \leq & -\chi_{2}V_{2}^{\frac{1}{2}} \end{array},$$

where

(28) $$\chi_{2}=\min\left\{D,\;E\right\}.$$

A positive constant $\Omega_{2}$ will satisfy the following inequality

(29) $$\Omega_{2}\leq \chi_{2}\left(t\right),\forall \psi <\left|s_{\theta }\left(t\right)\right|\leq \frac{\varepsilon }{2}.$$

With the integration of (27) and (29), we obtain

(30) $${\dot{V}}_{2}\leq -\Omega_{2}V_{2}^{\frac{1}{2}},$$

and the convergence time is calculated as

(31) $$t_{a}\leq \frac{V_{2}^{\frac{1}{2}}\left(\bar{t}\right)-V_{2}^{\frac{1}{2}}\left(\bar{t}+t_{a}\right)}{0.5\Omega_{2}}.$$

The finite-time convergence for $\left|s_{\theta }\left(\bar{t}\right)\right|>\psi$ is also guaranteed, i.e., $s_{\theta }\left(t\right)$ will reach the region [−ψ, ψ] in time $t_{a}$ from its initial value $s_{\theta }\left(\bar{t}\right)$.

Finally, $s_{\theta }$ will satisfy $\left|s_{\theta }\left(t\right)\right|\leq \psi <\varepsilon$ for all $t>\bar{t}+t_{a}$ under the proposed ABFSMC control laws (11)–(13). Hence, Case 2 is proven.

To summarize, the sliding mode variable $s_{\theta }$ will achieve $\left|s_{\theta }\left(t\right)\right|\leq \psi <\varepsilon$ in a finite time $\bar{t}+t_{a}$ under ABFSMC control laws (11)–(13). Furthermore, we can infer from (10) that $\left|\theta_{e}\right|\leq \psi <\varepsilon$. Finally, the proof of Theorem 1 is completed. □

**Remark 2.**  *For*$\left|s_{\theta }\right|$*with any initial value*$\left|s_{\theta }(0)\right|\geq \varepsilon /2$*at*t=0*,*$\hat{\eta }$*will keep increasing due to the integral of a positive function*$\left|s_{\theta }\right|$*according to (12). Until there exists a time*$t_{m}$*such that*$\hat{\eta }(t_{m})\geq \left|\varpi \right|$*, according to (14), it can be seen that*${\dot{s}}_{\theta }<0$*, which implies that*$\left|s_{\theta }\right|$*will start to decrease. Meanwhile,*$\hat{\eta }$*will keep increasing until*$\left|s_{\theta }(0)\right|=0$*in a finite-time period*Δt*. After that,*$\hat{\eta }$*will retain a final value*$\hat{\eta }(t_{m}+\Delta t)$*. It is obvious that*$\hat{\eta }(t_{m}+\Delta t)$*is finite due to its continuity property. This means that*$\hat{\eta }$*is always upper bounded by a positive number, say*$\eta_{2}$*, and also*$\eta_{2}\geq \left|\varpi \right|$.

**Remark 3.**  *According to (12), the designed ABFSMC control law (11) provides the sliding mode variable* $s_{\theta }$ *with a monotonically increasing gain to converge rapidly into the region* [−ε, ε] *when* $0\leq t\leq \bar{t}$ *. Meanwhile,* $L_{b}\left(s_{\theta }\right)$ *in the designed ABFSMC control law (11) will rapidly increase the adaptive error compensation* $\hat{\eta }$ *, such that the control input* ω *increases. Along this line, the sliding mode variable* $s_{\theta }$ *is promptly driven back to the origin when* $\left|s_{\theta }\right|$ *increases within* (0, ε) *and* $t>\bar{t}$ *. If* $\left|s_{\theta }\right|$ *decreases, then* $L_{b}\left(s_{\theta }\right)$ *will reduce the adaptive error compensation* $\hat{\eta }$ *accordingly to avoid overestimation of the disturbance compensation. This feature is the main benefit that BF provides.*

### 3.3. Position Controller of the Outer Loop

To guarantee the asymptotical convergence as well as boundedness and continuity of the convergence rate, the following sliding mode variable of the BTINTSMC is designed for the translational kinematics (9)

(32) $$s=e-e\left(0\right)+C\int_{0}^{t}\alpha \left(e\left(\tau \right)\right)d\tau ,$$

(33) $$\alpha \left(e\right)=\{\begin{array}{cc} \sin\left(\frac{\mathrm{sign}\left(e\right)\pi {\left|e\right|}^{\frac{q_{2}}{p_{2}}}}{2\xi^{\frac{q_{2}}{p_{2}}}}\right) & \left|e\right|\leq \xi \\ \mathrm{sign}\left(e\right) & \left|e\right|>\xi \end{array}.$$

where C is a positive constant coefficient, $p_{2}$ and $q_{2}$ are positive odd numbers, $1<p_{2}/q_{2}<2$, ξ is a positive constant parameter that represents the distance of the current state toward the desired trajectory, and e can be replaced by $x_{e}$ and $y_{e}$, then the respective sliding mode variables $s_{x}$ and $s_{y}$ can be obtained.

**Figure 4:**
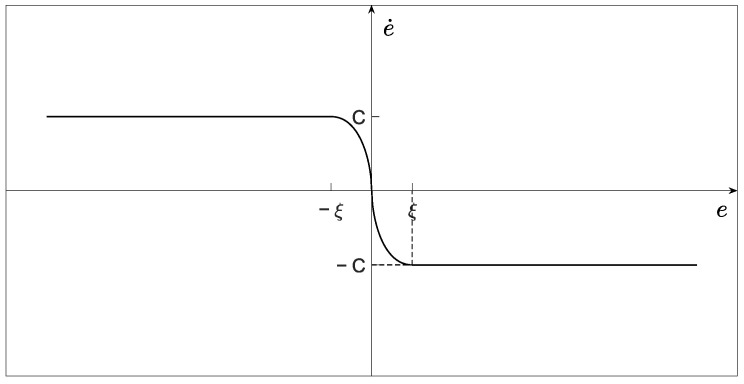
Sliding mode surface of the proposed BTINTSMC method.

With a predefined constant ξ, one can divide the whole tracking phase into two parts: full-speed convergence (|e|>ξ) and stable tracking (|e|≤ξ) phases. As illustrated in Figure 4, the system state converges to |e|=ξ at a constant speed and then smoothly switches to the stable tracking phase in the full-speed convergence phase. Meanwhile, the system state will converge to the origin in a finite time during the stable tracking phase.

**Theorem 2.**  *Consider the translational kinematics of (9) and the switching sliding mode surface (32). If the following control laws are adopted*

(34) $$u_{x}=v_{d}\cos\theta_{r}-k_{2}{\left|s_{x}\right|}^{\frac{q_{3}}{p_{3}}}\mathrm{erf}\left(hs_{x}\right)-C_{1}\alpha \left(x_{e}\right),$$

(35) $$u_{y}=v_{d}\sin\theta_{r}-k_{2}{\left|s_{y}\right|}^{\frac{q_{3}}{p_{3}}}\mathrm{erf}\left(hs_{y}\right)-C_{2}\alpha \left(y_{e}\right),$$

*where* $p_{3}$ *and* $q_{3}$ *are positive odd numbers,* $1<p_{3}/q_{3}<2$ *,* $k_{2}$ *and* h *are positive constant coefficients, and the positioning subsystem of the formulated NFMR system (9) is stabilized via the proposed BTINTSMC method.*

**Proof.**  First, a Lyapunov function is designed as

(36) $$V_{3}=\frac{1}{2}s_{x}^{2}+\frac{1}{2}s_{y}^{2}.$$

We obtain the derivative of the sliding functions $s_{x}$ and $s_{y}$ as

(37) $$\begin{array}{lll} {\dot{s}}_{x} & = & {\dot{x}}_{e}+C_{1}\alpha \left(x_{e}\right)\\ & = & u_{x}-v_{d}\cos\theta_{r}+C_{1}\alpha \left(x_{e}\right)\\ & = & v_{d}\cos\theta_{r}-k_{2}{\left|s_{x}\right|}^{\frac{q_{3}}{p_{3}}}\mathrm{erf}\left(hs_{x}\right)-C_{1}\alpha \left(x_{e}\right)-v_{d}\cos\theta_{r}+C_{1}\alpha \left(x_{e}\right)\\ & = & -k_{2}{\left|s_{x}\right|}^{\frac{q_{3}}{p_{3}}}\mathrm{erf}\left(hs_{x}\right) \end{array},$$

(38) $$\begin{array}{lll} {\dot{s}}_{y} & = & {\dot{y}}_{e}+C_{2}\alpha \left(y_{e}\right)\\ & = & u_{y}-v_{d}\sin\theta_{r}+C_{2}\alpha \left(y_{e}\right)\\ & = & v_{d}\sin\theta_{r}-k_{2}{\left|s_{y}\right|}^{\frac{q_{3}}{p_{3}}}\mathrm{erf}\left(hs_{y}\right)-C_{2}\alpha \left(y_{e}\right)-v_{d}\sin\theta_{r}+C_{2}\alpha \left(y_{e}\right)\\ & = & -k_{2}{\left|s_{y}\right|}^{\frac{q_{3}}{p_{3}}}\mathrm{erf}\left(hs_{y}\right) \end{array}.$$

The combination of (36)–(38) obtains

(39) $$\begin{array}{cc} {\dot{V}}_{3} & =s_{x}{\dot{s}}_{x}+s_{y}{\dot{s}}_{y}\\ & =s_{x}\left(-k_{2}{\left|s_{x}\right|}^{\frac{q_{3}}{p_{3}}}\mathrm{erf}\left(hs_{x}\right)\right)+s_{y}\left(-k_{2}{\left|s_{y}\right|}^{\frac{q_{3}}{p_{3}}}\mathrm{erf}\left(hs_{y}\right)\right)\\ & \leq -k_{2}{\left|s_{x}\right|}^{\frac{q_{3}}{p_{3}}+1}{\left(\mathrm{erf}\left(hs_{x}\right)\right)}^{2}-k_{2}{\left|s_{y}\right|}^{\frac{q_{3}}{p_{3}}+1}{\left(\mathrm{erf}\left(hs_{y}\right)\right)}^{2} \end{array}$$

It is concluded that ${\dot{V}}_{3}<0$ when $\left|s_{x}\right|\neq 0$ and $\left|s_{y}\right|\neq 0$, thereby indicating that system states can reach their corresponding sliding mode surface and maintain it under the proposed control laws (34) and (35). According to Figure 4, $\mathrm{sign}({\dot{x}}_{e})=-\mathrm{sign}(x_{e})$ and $\mathrm{sign}({\dot{y}}_{e})=-\mathrm{sign}(y_{e})$ exist on the sliding mode surface, which implies that $x_{e}$ and $y_{e}$ will converge to zero. Hence, the proof is completed. □

We obtain the following according to (34) and (35):

(40) $$\begin{array}{lll} \theta_{d} & = & \mathrm{atan}2\left(\frac{u_{y}}{u_{x}}\right)\\ & = & \mathrm{atan}2\left(\frac{v_{d}\sin\theta_{r}-k_{2}{\left|s_{y}\right|}^{\frac{q_{3}}{p_{3}}}\mathrm{erf}(hs_{y})-C_{2}\alpha }{v_{d}\cos\theta_{r}-k_{2}{\left|s_{x}\right|}^{\frac{q_{3}}{p_{3}}}\mathrm{erf}(hs_{x})-C_{1}\alpha }\right) \end{array}.$$

**Remark 4.**  *It is noted that* s(0)=0 *implies that system states are on the sliding mode surface (32) from the initial moment. System states can theoretically be maintained on sliding mode surfaces from beginning to end. Given this context, the virtual inputs* $u_{x}$ *and* $u_{y}$ *can be retained in the bounded range determined by custom coefficients* $C_{i=1,\;2}$ *. According to* $v=\sqrt{u_{x}^{2}+u_{y}^{2}}$ *,* v *is bounded by selecting the appropriate* $\left\{k_{2},\;p_{2,3},\;q_{2,3},C_{1,2}\right\}$ *. Moreover, we can also set different proportional relationships of* $C_{1}$ *and* $C_{2}$ *to allow the NFMR to go back to the desired trajectory in different ways from the initial position, according to (40).*

**Remark 5.**  *The employment of switching term* sign(⋅) *in conventional SMC methods allows signal variables to arrive at specified coupled sliding mode surfaces. However, the DLSMC mechanism requires sufficient continuity. Therefore, the* sign(⋅) *function is replaced by the* erf(⋅) *in this study to ensure smooth switching of the sliding mode and solve the discontinuity problem.*

### 3.4. Stability Analysis

**Theorem 3.**  *When control laws (11), (34), and (35) are adopted under switching functions (10) and (32), the proposed DLSMC mechanism will stabilize the formulated NFMR kinematics, i.e., the tracking errors will converge to zero.*

**Proof.**  Integrating the proposed cascade control mechanism allows us to reformulate (9) as

(41) $$\begin{array}{l} {\dot{x}}_{e}=-k_{2}{\left|s_{x}\right|}^{\frac{q_{3}}{p_{3}}}\mathrm{erf}(hs_{x})-C_{1}\alpha \left(x_{e}\right)\\ {\dot{y}}_{e}=-k_{2}{\left|s_{y}\right|}^{\frac{q_{3}}{p_{3}}}\mathrm{erf}(hs_{y})-C_{2}\alpha \left(y_{e}\right)\\ {\dot{\theta }}_{e}=-k_{1}s_{\theta }-\hat{\eta }\mathrm{sign}\left(s_{\theta }\right)+\varpi \end{array}.$$

According to Theorem 1, $\theta_{e}(t)$ converges to the small area ε in a finite time $t_{1}=\bar{t}+t_{a}$ for any given initial state $\theta_{e}(0)$. Hence, system error states $x_{e}$ and $y_{e}$ will detach from sliding mode surfaces until $t_{1}$. In this way, $s_{x}(t_{1})$ and $s_{y}(t_{1})$ are then guaranteed to be bounded. When h is selected as a large enough number, we can approximatively obtain the following according to (37) and (38)

(42) $${\dot{s}}_{x}=-k_{2}{\left|s_{x}\right|}^{\frac{q_{3}}{p_{3}}}\mathrm{erf}\left(hs_{x}\right)=-k_{2}{\left|s_{x}\right|}^{\frac{q_{3}}{p_{3}}}\mathrm{sign}\left(s_{x}\right),$$

(43) $${\dot{s}}_{y}=-k_{2}{\left|s_{y}\right|}^{\frac{q_{3}}{p_{3}}}\mathrm{erf}\left(hs_{y}\right)=-k_{2}{\left|s_{y}\right|}^{\frac{q_{3}}{p_{3}}}\mathrm{sign}\left(s_{y}\right).$$

Furthermore, the convergence time of $s_{x}$ and $s_{y}$ can be approximatively obtained by the following formulas

(44) $$t_{x}^{r}=\frac{p_{3}}{k_{2}\left(p_{3}-q_{3}\right)}{\left|s_{x}\left(t_{1}\right)\right|}^{1-\frac{q_{3}}{p_{3}}},$$

(45) $$t_{y}^{r}=\frac{p_{3}}{k_{2}\left(p_{3}-q_{3}\right)}{\left|s_{y}\left(t_{1}\right)\right|}^{1-\frac{q_{3}}{p_{3}}}.$$

By defining $\tilde{t}=\max\left\{t_{x}^{r}+t_{1},\;t_{y}^{r}+t_{1}\right\}$, the orientation kinematics will converge to the origin, and the translational kinematics will reach the sliding mode surfaces within $t>\tilde{t}$. Then the error state kinematics (41) is simplified as

(46) $${\dot{x}}_{e}=-C_{1}\alpha \left(x_{e}\right),$$

(47) $${\dot{y}}_{e}=-C_{2}\alpha \left(y_{e}\right).$$

We first consider the $x_{e}$ subsystem. As described above, the system error state $x_{e}$ is on the sliding mode surface (32) when $t>\tilde{t}$. Since two situations exist, $\left|x_{e}(\tilde{t})\right|>\xi$ or $\left|x_{e}(\tilde{t})\right|<\xi$, the proof is divided into two cases.

Case (1): Under BTINTSMC law (34) for $\left|x_{e}\right|>\xi$

The Lyapunov candidate function $V_{4}$ is defined as

(48) $$V_{4}=\frac{1}{2}{\left(\left|x_{e}\right|-\xi \right)}^{2}.$$

Based on (33), the derivative of $V_{4}$ along (46) satisfies the following

(49) $$\begin{array}{cc} {\dot{V}}_{4} & =\left(\left|x_{e}\right|-\xi \right){\dot{x}}_{e}\mathrm{sign}\left(x_{e}\right)\\ & =-C_{1}\alpha \left(x_{e}\right)\mathrm{sign}\left(x_{e}\right)\left(\left|x_{e}\right|-\xi \right)\\ & =-C_{1}\left(\left|x_{e}\right|-\xi \right)\\ & =-\sqrt{2}C_{1}V_{{}_{4}}^{\frac{1}{2}} \end{array}$$

We can then obtain the following

(50) $$t_{x}^{b}\leq \sqrt{2}\frac{V_{4}^{\frac{1}{2}}\left(\tilde{t}\right)-V_{4}^{\frac{1}{2}}\left(\tilde{t}+t_{x}^{b}\right)}{C_{1}}.$$

Therefore, from the moment it reaches the sliding mode surface, $x_{e}(t)$ will converge to the region of [−ξ, ξ] after a finite time $t_{x}^{b}$ when $\left|x_{e}\right|>\xi$. Then, for all $t>t_{x}^{b}+\tilde{t}$, one can conclude that $\left|x_{e}\right|<\xi$, and the control law will be turned into the second equation in (33). Considering that, $x_{e}$ will converge to the range [−ξ, ξ] under control laws (33) and (34) in the finite time $t_{x}^{b}+\tilde{t}$.

Case (2): Under the BTINTSMC law (34) for $\left|x_{e}\right|\leq \xi$

The Lyapunov candidate function $V_{5}$ is defined as follows

(51) $$V_{5}=\frac{1}{2}x_{e}{}^{2}.$$

The derivative of $V_{5}$ along (46) satisfies the following using (33)

(52) $${\dot{V}}_{5}=x_{e}{\dot{x}}_{e}=-C_{1}x_{e}\alpha \left(x_{e}\right)=-C_{1}x_{e}\sin\left(\frac{\mathrm{sign}\left(x_{e}\right)\pi {\left|x_{e}\right|}^{\frac{q_{2}}{p_{2}}}}{2\xi^{\frac{q_{2}}{p_{2}}}}\right)\leq -\frac{C_{1}}{\xi^{\frac{q_{2}}{p_{2}}}}{\left|x_{e}\right|}^{\frac{q_{2}}{p_{2}}+1}.$$

We can then obtain the following equation

(53) $$t_{x}^{c}=\frac{p_{2}}{\frac{C_{1}}{\xi^{\frac{q_{2}}{p_{2}}}}\left(p_{2}-q_{2}\right)}{\left|x_{e}\left(t_{x}^{b}+\tilde{t}\right)\right|}^{1-\frac{q_{2}}{p_{2}}}.$$

It should be mentioned that $x_{e}$ will clearly converge to zero under control laws (33) and (34) in a finite time when the system state is reached on the sliding mode surface and $\left|x_{e}\right|\leq \xi$. Hence, we have $\left|x_{e}(t)\right|=0$ for all $t>\tilde{t}+t_{x}^{b}+t_{x}^{c}$.

For the $y_{e}$ subsystem in (46), by choosing

(54) $$V_{6}=\frac{1}{2}{\left(y_{e}-\xi \right)}^{2},\;V_{7}=\frac{1}{2}y_{e}{}^{2},$$

we obtain the following

(55) $$t_{y}^{b}\leq \sqrt{2}\frac{V_{6}^{\frac{1}{2}}\left(\tilde{t}\right)-V_{6}^{\frac{1}{2}}\left(\tilde{t}+t_{y}^{b}\right)}{C_{2}},$$

(56) $$t_{y}^{c}=\frac{p_{2}}{\frac{C_{2}\pi }{2\xi^{\frac{q_{2}}{p_{2}}}}\left(p_{2}-q_{2}\right)}{\left|y_{e}\left(t_{1}+t_{y}^{b}\right)\right|}^{1-\frac{q_{2}}{p_{2}}}.$$

The double-loop control system will stay at the origin for all $t>t_{end}$ and $t_{end}=\max\left\{t>\tilde{t}+t_{x}^{b}+t_{x}^{c},\;t>\tilde{t}+t_{y}^{b}+t_{y}^{c}\right\}$. Finally, the proof of Theorem 3 is completed. □

**Remark 6.**  *The time*$\tilde{t}$*should be as short as possible to guarantee the convergence of the DLSMC mechanism. Two methods can be applied to achieve this goal. First, selecting proper coefficients*$\left\{k_{2},\;p_{2,3},\;q_{2,3}\right\}$*of the sliding mode variable*$s_{x,y}$*to obtain a proper convergence rate slows down not only the growth of*$s_{x,y}$*in*$t_{1}$*but also accelerates the convergence of*$s_{x,y}$*in*$\tilde{t}$*. Second, convergence time*$t_{1}$*can be reduced by increasing*$k_{1}$*to enhance the convergence rate of the inner loop. The specific parameter selection process will be discussed in the next section. Meanwhile, the appropriate selection of*$\left\{k_{1,2},\;p_{1,2,3},\;q_{1,2,3},C_{1,2}\right\}$*also can satisfy the constraints of*u(t).

## 4. Validation and Results

### 4.1. Overall Control Mechanism

**Figure 5:**
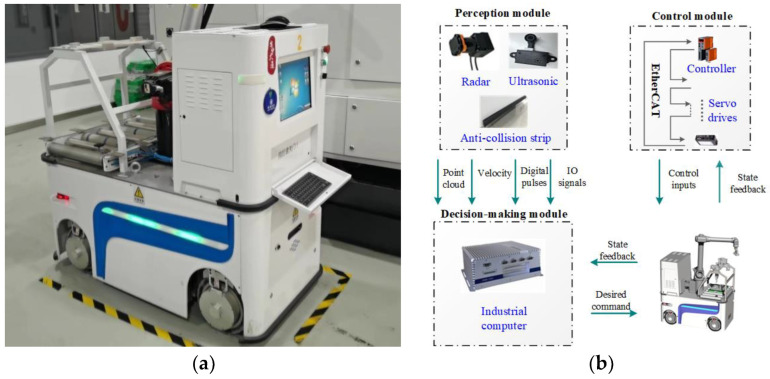
Experimental platform. (**a**) The developed NFMR. (**b**) Hardware architecture.

**Figure 6:**
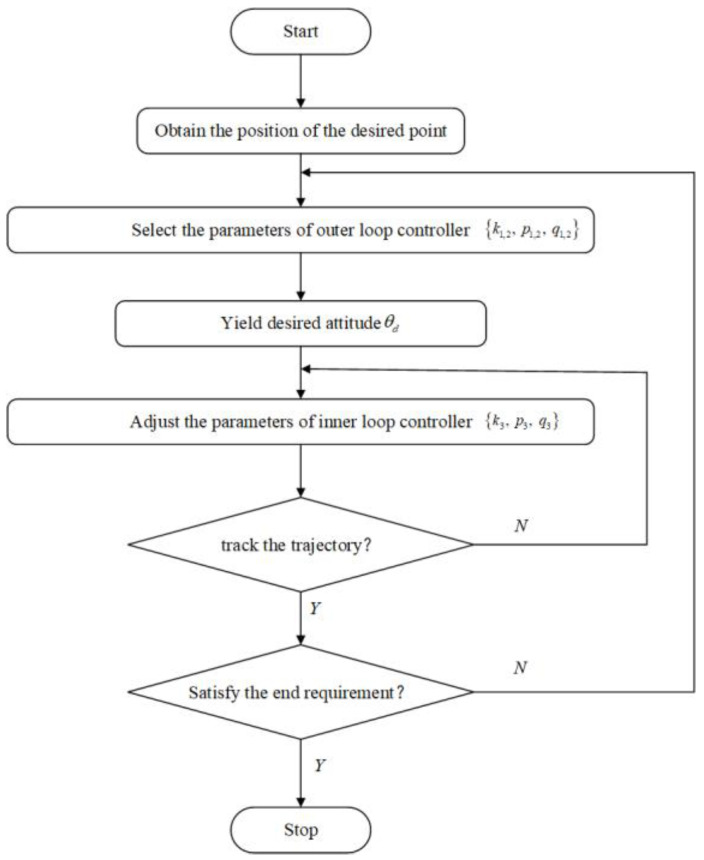
Implementation procedure of the proposed mechanism.

Figure 5a shows the developed four-wheel NFMR with independent steering and drive. The NFMR can switch between the conventional Ackerman and double-Ackerman steer modes due to the steering maneuverability of each wheel. The main specifications of the NFMR are listed in Table 1. As shown in Figure 5b, the hardware architecture of the NFMR consists of the following modules: (1) perception (date of environment detection, map construction, and state feedback can be obtained with the help of various installed sensors), (2) decision-making (reaction of the NFMR is determined according to the received data and information), and (3) control (enabling the NFMR to complete the tracking task) layers. The implementation procedure is presented in Figure 6.

The following parameters of the NFMR are specified: (1) parameters of control laws (31) and (32), $q_{1}=23$, $q_{2,3}=17$, $p_{1,2,3}=33$, ξ=0.5, $C_{1,2}=0.6$, $k_{1,2,3}=1$, $L_{0}=\lambda =1$, $L_{1}=1.2$, and ε=0.01; (2) input limitations $v_{\min}=0.2\;m/s$, $v_{\max}=2\;m/s$, and $\omega_{\max}=1.59\;\mathrm{rad}/s$. A control sample time of 1 ms is applied to implement the developed NFMR system. 

The experiment will be divided into two cases to verify the tracking ability of the trajectory with the existence of large initial state errors and compare the robustness of each control method. Case 1 highlights the advantages of the proposed DLSMC mechanism. We adopt the following methods for comparison: (i) Single loop methods include traditional PID (for designing the yaw angle rate input with $y_{e}$ and $\theta_{e}$; with constant velocity), couple CSMC (for designing the couple sliding mode surface of $y_{e}$ and $\theta_{e}$) and decouple DSMC (with the pseudoinverse method) SMCs [15]; (ii) Double loop mechanisms, including double constant rate SMC (DL-CRSMC) [19], double nonsingular terminal SMC (DNTSMC) [21], and bounded time-varying SMC (BTSMC) in the outer loop with NTSMC in the inner loop which is derived through the proposed control principle. Case 2 highlights the advantages of the proposed inner loop ABFSMC. We only replace different inner loop controllers for comparison and add the disturbance at the fifth and fiftieth seconds. Meanwhile, the control group similar to Case 1 is used in this case, but the outer loop controller of each double loop method shall be replaced with the same controller to obtain the same desired yaw angle determined by the outer loop controller. All the controllers are tuned optimally with the same initial state of the considered robot, and the simulation experiments are performed under the same operating conditions. Specifically, the parameter acquisition of the DNTSMC control method without the consideration of the anti-disturbance mechanism and the DL-BTSMC control method with the NTSMC controller in the inner loop is the same as that of the proposed method. The parameter acquisition of the DCRSMC control method is obtained by referring to the corresponding literature [19]. When it comes to the PID, CSMC, and DSMC controller, the stabilization of parameters is realized using the natural-inspired optimization algorithm named artificial bee colony [35]. The fixed gain should consider tracking performance and interference suppression, and the gain is obtained by pre-tuning with a weight function.

### 4.2. Results and Discussions

*Case (1) Global tracking performance with large initial error*

**Figure 7:**
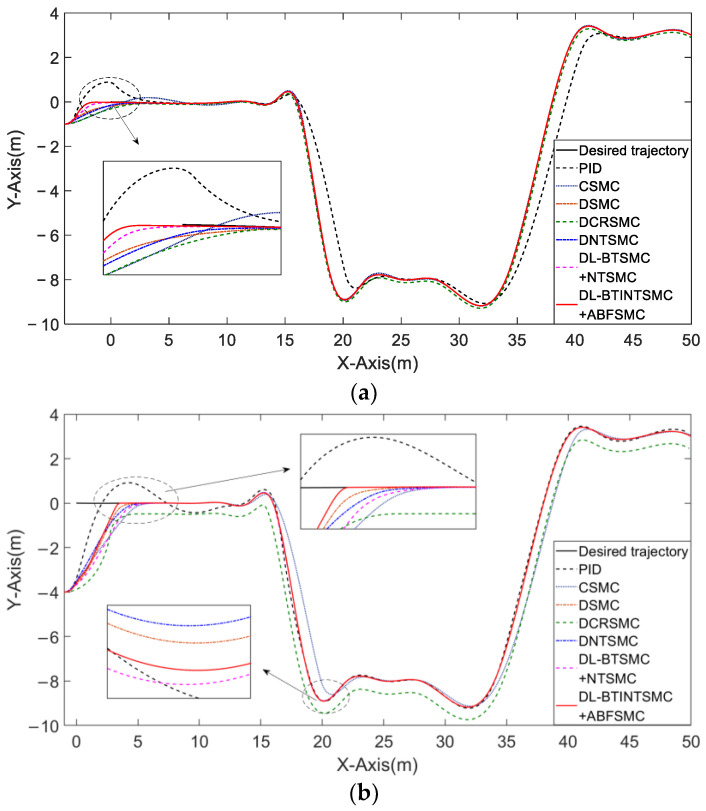
The comparison of trajectory following the performance of different control mechanisms. (**a**) a large lateral initial error; (**b**) a large longitudinal initial error.

The NFMR is controlled in Case 1 to track continuous profiles with bounded input signals beginning at a reasonable distance from the trajectory. Figure 7 presents the comparison of the tracking performance and convergence of the position error in the two situations. As shown in Figure 7a, the initial states of the NFMR in the first situation are set to ${\left(x(0),\;y(0),\;\theta (0)\right)}^{T}={\left(-4,-1,\;0\right)}^{T}$, which demonstrates a large lateral initial error. We adopt the PID and CSMC methods in this situation, and the overshoot occurs in the initial state error convergence stage. Notably, the CSMC method exhibits poor tracking of long-distance longitudinal trajectories. As expected, the proposed DLSMC mechanism showed the optimal tracking performance in either the initial state error convergence or tracking the desired trajectory. As shown in Figure 7b, initial states of the NFMR in the other situation are set to ${\left(x(0),\;y(0),\;\theta (0)\right)}^{T}={\left(-1,-4,\;0\right)}^{T}$, which demonstrates a large longitudinal initial error. The DSMC method fails to control the NFMR reaching the trajectory because the lateral error converges to zero earlier than the longitudinal error $x_{e}$ and then interrupts the convergence of $y_{e}$. Furthermore, DLSMC mechanisms exhibit better tracking performance than single-loop ones.

**Figure 8:**
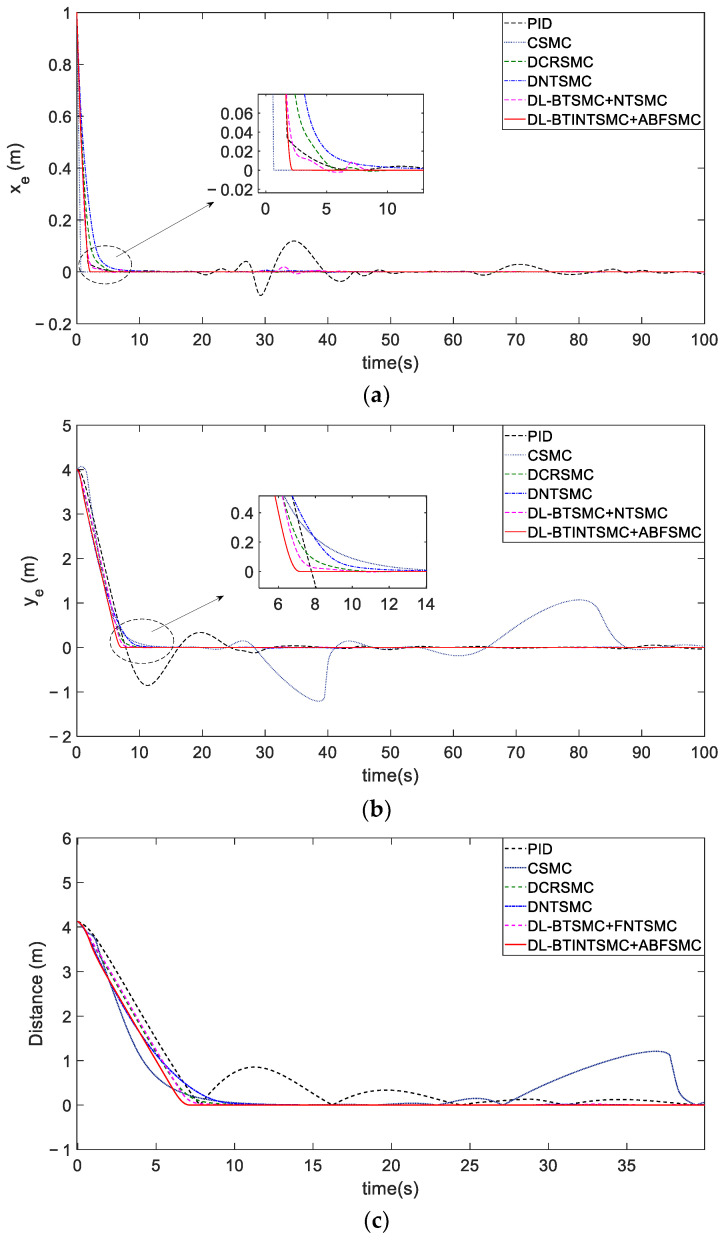
The comparison of tracking errors of different control mechanisms. (**a**) process of longitudinal error convergence. (**b**) process of lateral error convergence. (**c**) process of position error convergence.

We select the second situation for in-depth analysis to highlight the comparison of tracking performance. Note that the DSMC method was not selected because it fails to track the desired trajectory. Figure 8 illustrates the tracking performance of each state with different control mechanisms. The proposed mechanism demonstrates optimal performance in terms of tracking error elimination and convergence. Compared with other methods, the proposed DLSMC mechanism can rapidly drive the NFMR back to the desired trajectory. Compared with the double loop mechanism, the PID method will lead to significant overshoots and show poor tracking accuracy in subsequent stages. However, the CSMR method presents a slightly improved initial error convergence and fast convergence of the lateral error (Figure 8a). This method is sensitive to trajectory curvature and exhibits a significantly slower convergence of longitudinal error (Figure 8b) that leads to poor convergence of comprehensive position error (Figure 8c). Notably, the convergence time of CSMC, DCRSMC, DNTSMC, the proposed BTSMC with NTSMC, and the proposed BTINTSMC with ABFSMC, except the PID method because it fails to maintain the required accuracy in tracking (distance of less than or equal to 0.001 m), is 18.8, 10.8, 21.7, 10, and 7.1 s, respectively. This finding validates the advantages of the novel DLSMC mechanism in error convergence with large initial state errors.

The corresponding quantitative criteria of position tracking accuracy are listed in Table 2 to illustrate the tracking performance of the presented DLSMC mechanism clearly. Our method can achieve enhanced performance criteria in terms of integral absolute error (IAE), integrated absolute error (ISE), and standard deviation (STD) of tracking errors. The proposed DLSMC mechanism shows enhanced dynamic control capacities and system robustness while guaranteeing satisfactory tracking performance for the developed NFMR in a comprehensive manner due to the coordination and complementarity of two different novel control methods in the inner and outer loops. Specifically, the proposed DLSMC mechanism achieves the best accuracy of 13.5757 m⋅s in terms of the IAE criterion, which is an improvement of 64.92% compared with that of the traditional PID method. Our control method also shows the minimum IAE and STD during the whole trajectory tracking. This finding implied that the proposed DLSMC mechanism provides the optimal transient response and the minimum error fluctuation around zero.

**Figure 9:**
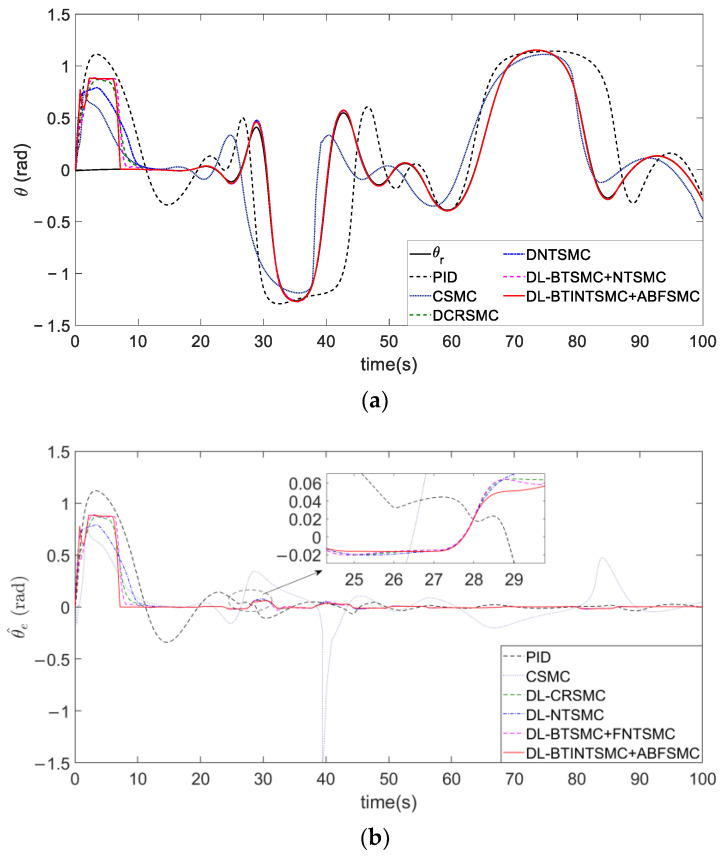
The comparison of tracking $\theta_{r}$. (**a**) tracking process. (**b**) error convergence.

**Figure 10:**
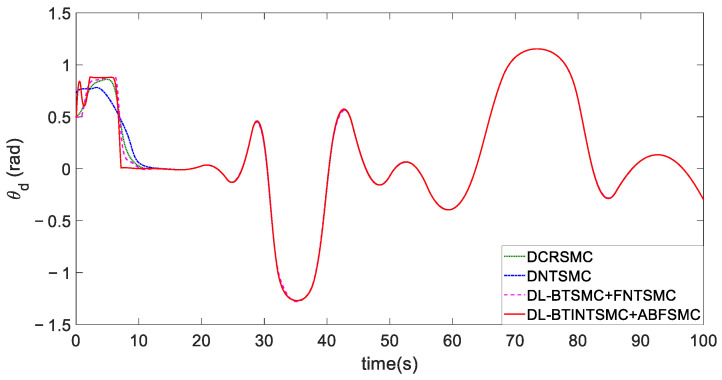
The desired yaw angle under different outer loop controllers.

Figure 9 illustrates the performance of real attitude tracking. $\theta_{r}$ is the tangent direction of the desired trajectory and $\theta_{r}=\mathrm{atan}2\left(dg(x)/dx\right)$. The presented DLSMC mechanism shows satisfactory dynamic performance. As shown in Figure 9a, double-loop control mechanisms clearly exhibit better convergence and stability than single-loop ones. The proposed DLSMC mechanism can induce the attitude of the NFMR to change more and faster in the initial stage and ensure its faster response to position error convergence than other double-loop control mechanisms. Moreover, the double-loop control mechanism, especially the presented DLSMC mechanism, presents stable and accurate tracking performance in the follow-up tracking process despite fluctuations in the curvature of the desired trajectory. The intuitive presentation in Figure 9b illustrates that the convergence time of CSMC, DCRSMC, DNTSMC, the proposed BTSMC with NTSMC, and the proposed BTINTSMC with ABFSMC is 15.1, 13, 16, 13, and 7.2 s, respectively. The comparison of the position error mentioned above and the real attitude error demonstrated that using the proposed mechanism, the convergence time of their position and real attitude error is basically very similar. However, attitude tracking is first ignored because of the presence of large initial state errors. Meanwhile, Figure 10 shows that the desired attitude $\theta_{d}$ is also different due to different outer loop controllers. Therefore, the evaluation of attitude tracking performance will be carried out in the next case.

**Figure 11:**
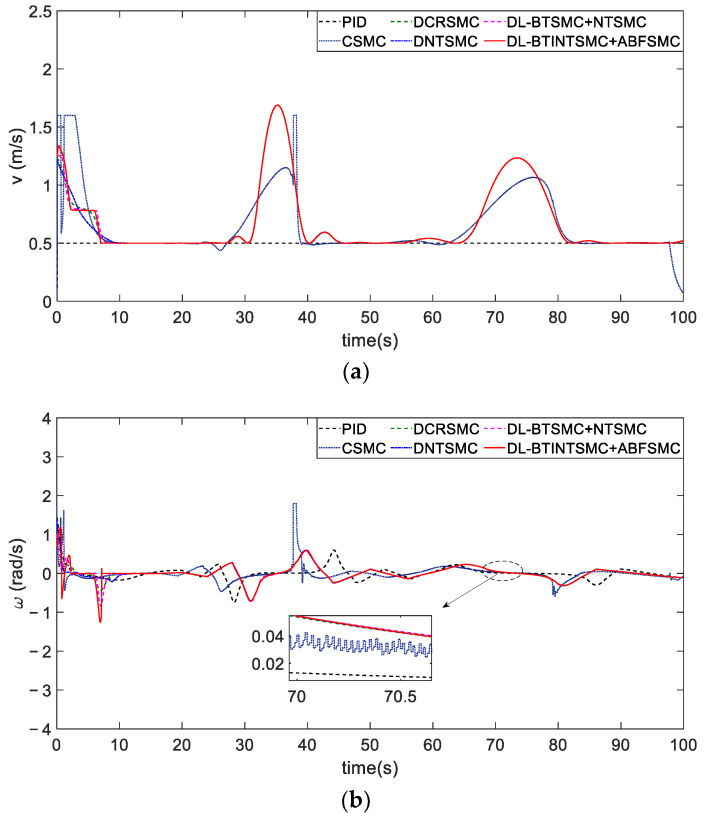
The velocity tracking responses in scenario 1. (**a**) the linear velocity control input of each controller. (**b**) the yaw angle rate control input of each controller.

**Figure 12:**
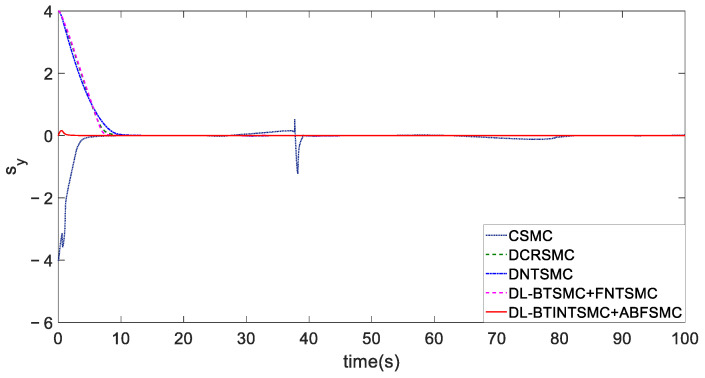
Sliding mode surfaces of $y_{e}$.

The velocity limitation is satisfactory under any control method (Figure 11a), but the yaw angle rate fails to meet the limitation while presenting violent chatter when the CSMC method is adopted (Figure 11b). Figure 12 illustrates the better sliding performance of the proposed BTINTSMC compared with that of outer loop controllers of other DLSMC mechanisms. The longitudinal error control is utilized as an example because the presented BTINTSMC is simultaneously used to control the convergence of longitudinal and transverse errors. Although the longitudinal sliding mode variable $s_{y}$ deviates from the sliding mode surface because the yaw angle θ has not tracked the desired angle $\theta_{d}$, $s_{y}$ only increases to a small value and then converges to zero in a short time. Especially, the convergence time of $s_{y}$ in CSMC, CRSMC, NTSMC, BTSMC, and BTINTSMC methods are 12.1, 10.8, 21.7, 10, and 6.2 s, respectively, which validates the advantages of presented TISS. The fast and smooth response of the outer loop controller with the BTINTSMC method to the reaching phase of the initial state helps achieve improved attitude-tracking performance.

**Figure 13:**
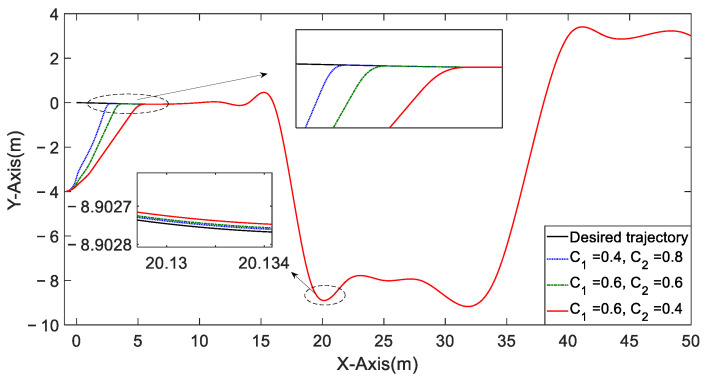
The tracking trajectory with different proportions of $C_{1,2}$.

Figure 13 shows the influence of the adjustment parameter $C_{1,2}$ on the convergence of initial state errors. The presented DLSMC mechanisms can adjust the initial state error convergence process freely by easily adjusting the ratio of $C_{1,2}$ to adapt to different initial states compared with single-loop control mechanisms.

*Case (2) Tracking performance of the inner loop controller with disturbances*

**Figure 14:**
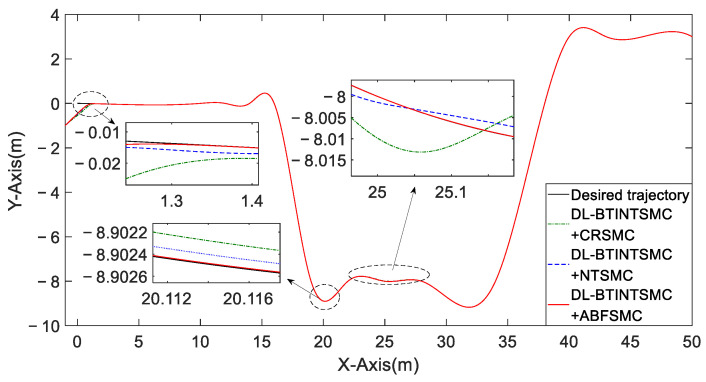
The comparison of trajectory following the performance of different inner loop controllers.

We use the same controller in the outer loop and then choose different control methods, including CRSMC, NTSMC, and ABFSMC, in the inner loop in Case 2. Initial states are then set to ${\left(x(0),\;y(0),\;\theta (0)\right)}^{T}={\left(-1,-1,\;\pi /6\right)}^{T}$. On the one hand, using different methods, the convergence trajectories error is similar in the initial stage, but using the ABFSMC method accelerates and stabilizes the convergence trajectory when reaching the desired trajectory (Figure 14). On the other hand, the proposed method shows the optimal tracking performance despite the presence of uncertainties and disturbances. 

**Figure 15:**
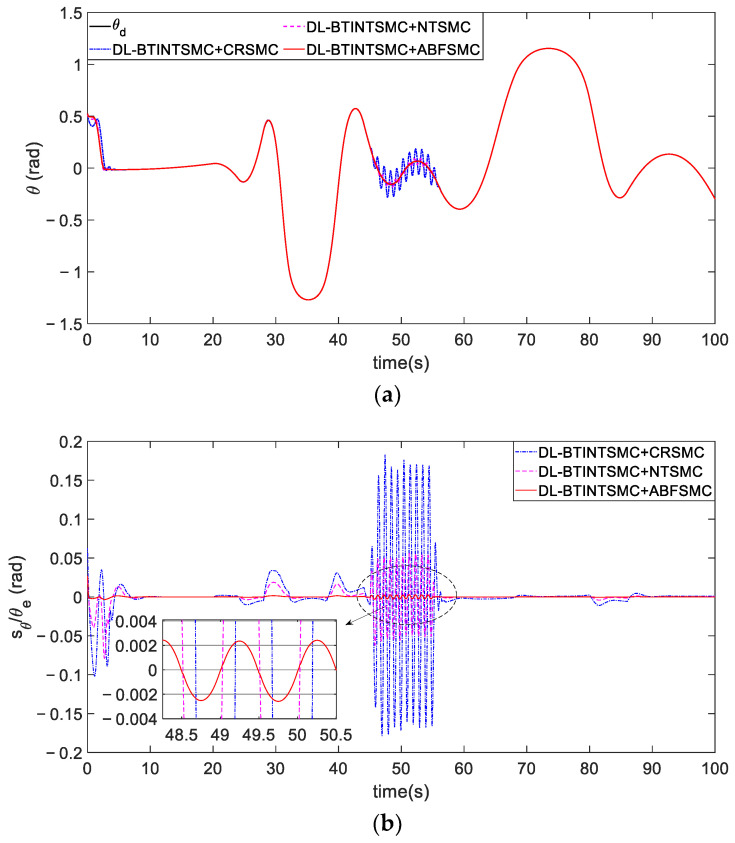
The comparison of tracking $\theta_{d}$. (**a**) tracking process. (**b**) error convergence.

Figure 15 shows the tracking of the desired attitude obtained via the outer loop. Tracking trajectories of the attitude are depicted in Figure 15a. Figure 15b presents that the attitude error $\theta_{e}$ (also the sliding mode variable $s_{\theta }$) of the presented ABFSMC method converges into a predefined bound $\left|s_{\theta }(t)\right|=\left|\theta_{e}(t)\right|<\varepsilon =0.01$ and is properly constrained by this bound despite external disturbances in the fifth and fiftieth seconds. Other methods induce $\theta_{e}$ to converge to a range of about ±0.18 rad. By contrast, the proposed method further mitigates the range to $\pm 3\times 10^{-3}\;\mathrm{rad}$ and considerably reduces the range by 99.98%. The quantitative criteria of $\theta_{e}$ listed in Table 3 are used to evaluate the contrastive performance clearly. The proposed method can effectively eliminate chattering, improve system robustness, and guarantee satisfactory tracking performance due to the strong constraint of the barrier function. Specifically, the yaw angle error achieves the optimal accuracy of 0.0001 rad⋅s in terms of the ISE criterion when the proposed ABFSMC is used in the inner loop. This value shows a maximum performance improvement of 99.9% compared with other methods. Furthermore, the ABFSMC method achieves the minimum IAE and STD values.

**Figure 16:**
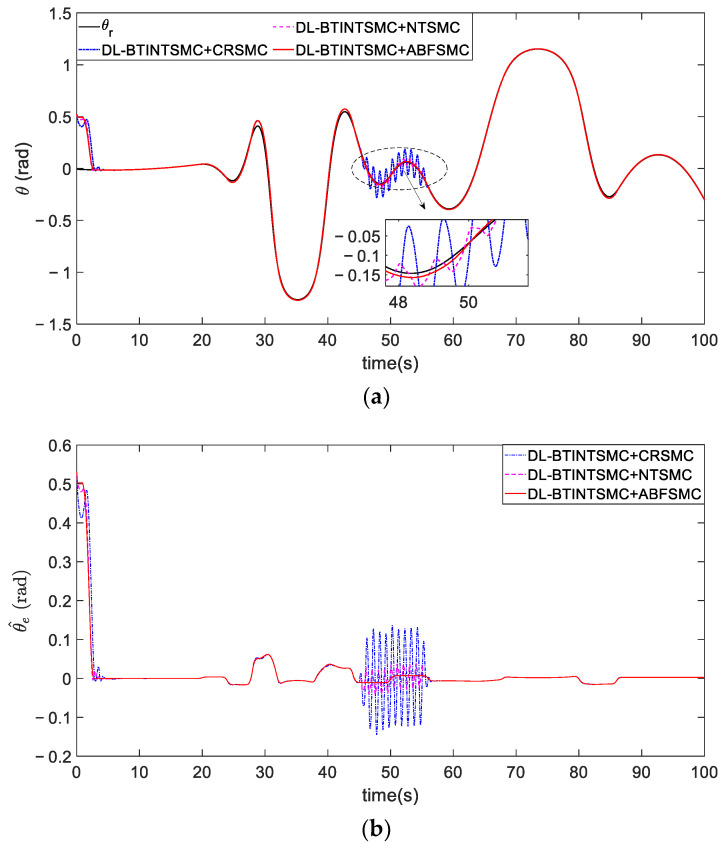
The comparison of tracking $\theta_{r}$. (**a**) tracking process. (**b**) error convergence.

Figure 16 illustrates the tracking of the real attitude of the desired trajectory. The process of attitude tracking is highly smooth and stable in the initial stage despite the presence of disturbance when the ABFSMC method is adopted. Optimal data of the quantitative criteria of the real attitude error ${\hat{\theta }}_{e}$ using the ABFSMC method are presented in Table 4. The ABFSMC method demonstrates a performance improvement of 32.4% compared with other methods.

**Figure 17:**
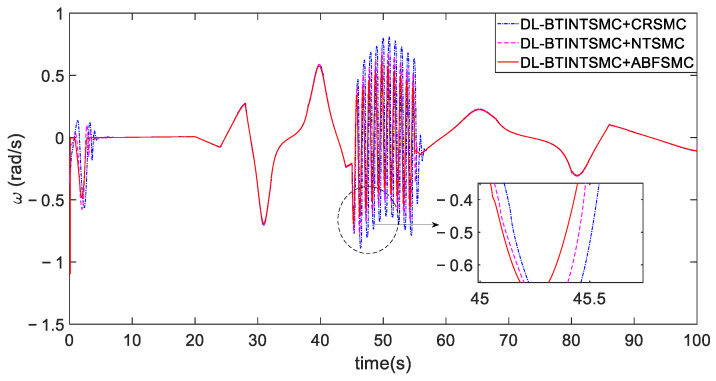
The comparison of yaw angle rate of different control mechanism.

As illustrated in Figure 17, adopting any control method, all the yaw angle rates satisfy the constraint input, |ω|≤1.59 rad/s. The proposed ABFSMC method can effectively restrain the influence of disturbance because it presents a smaller yaw angle rate variation amplitude and faster response speed to the disturbance than other methods in the case of external disturbance.

## 5. Conclusions

An enhanced DLSMC mechanism for the NFMR control with BTINTSMC as the outer loop controller and ABFSMC as the inner loop controller was proposed in this work. The BTINTSMC allows the position states of NFMR to start from the sliding mode surface for the outer loop to almost eliminate the reaching phase and enhance the robustness. The NFMR can return to the desired trajectory under a bounded input by adjusting several simple parameters. The ABFSMC method can improve the stability of the closed-loop process because it maintains the attitude state of the NFMR in a predefined small area near the sliding mode surface for the inner loop to eliminate chattering caused by uncertainties and external disturbances. The stability and convergence were analyzed. The validation results are consistent with those of the theoretical analysis in terms of trajectory tracking, thereby indicating the feasibility and superiority of the proposed DLSMC mechanism. It is shown that our method ensures faster convergence, shorter distance, and more stable tracking compared with existing methods.

For the proposed DLSMC mechanism, we can conduct further research in the following aspects: (1) designing the adaptive adjustment method of the coefficients of BTINTSMC so that the NFMR can return to the desired trajectory with the optimate trajectory in any initial state; (2) applying this proposed DLSMC mechanism for cluster control of mobile robots.

## Figures and Tables

**Table 1:** The main specifications of the NFMR.

Parameters	Value	Parameters	Value
Length	0.89 m	Incremental encoder	2500 ppr
Width	0.53 m	Frequency of PC	2.59 GHz
Wheel diameter	0.28 m	RAM of PC	8 G
Total weight	1 t	Max speed	2 m/s
LiDAR sweep distance	60 m	Battery run-time	8 h
Rated torque of the driving/steering motor	20 Nm	Rated power of the driving/steering motor	1.13 kw

**Table 2:** Position tracking error performance criteria of different controllers.

Error	Criterion	PID	CSMC	DCRSMC	DNTSMC	BTSMC + NTSMC	BTINTSMC + ABFSMC
Distance	IAE	34.1069	38.7033	15.1050	15.0703	15.2021	13.5757
	ISE	70.4731	54.9742	41.1628	38.0608	42.1426	37.1057
	STD	0.6769	0.6335	0.6235	0.5982	0.6311	0.5938

**Table 3:** $\theta_{d}$-tracking error performance criteria of different inner loop controllers.

Criterion	BTINTSMC + CRSMC	BTINTSMC + NTSMC	BTINTSMC + ABFSMC
IAE	1.6398	0.5831	0.0403
ISE	0.1592	0.0205	0.0001
STD	0.0399	0.0143	0.0010

**Table 4:** $\theta_{r}$-tracking error performance criteria of different control mechanisms.

Criterion	BTINTSMC + CRSMC	BTINTSMC + NTSMC	BTINTSMC + ABFSMC
IAE	2.5522	1.8415	1.7249
ISE	0.5341	0.4463	0.4402
STD	0.0721	0.0659	0.0654

## Data Availability

Not applicable.

## References

[1] Zhang X., Huang Y., Wang S., Meng W., Li G., Xie Y. (2021). Motion planning and tracking control of a four-wheel independently driven steered mobile robot with multiple maneuvering modes. Front. Mech. Eng..

[2] Morin P., Samson C., Siciliano B., Khatib O. (2008). Motion control of wheeled mobile robots. Handbook of Robotics.

[3] Campion G., Bastin G., Dandrea-Novel B. (1996). Structural properties and classification of kinematic and dynamic models of wheeled mobile robots. IEEE Trans. Robot. Autom..

[4] Park B.S., Yoo S.J., Park J.B., Choi Y.H. (2010). A Simple Adaptive Control Approach for Trajectory Tracking of Electrically Driven Nonholonomic Mobile Robots. IEEE Trans. Control Syst. Technol..

[5] Wang Z., Li G., Chen X., Zhang H., Chen Q. (2019). Simultaneous Stabilization and Tracking of Nonholonomic WMRs with Input Constraints: Controller Design and Experimental Validation. IEEE Trans. Ind. Electron..

[6] Fierro R., Lewis F.L. (1997). Control of a nonholomic mobile robot: Backstepping kinematics into dynamics. J. Robot. Syst..

[7] Fukao T., Nakagawa H., Adachi N. (2000). Adaptive tracking control of a nonholonomic mobile robot. IEEE Trans. Robot. Autom..

[8] Lian C., Xu X., Chen H., He H. (2016). Near-Optimal Tracking Control of Mobile Robots Via Receding-Horizon Dual Heuristic Programming. IEEE Trans. Cybern..

[9] Li Z., Yang C., Su C.-Y., Deng J., Zhang W. (2015). Vision-Based Model Predictive Control for Steering of a Nonholonomic Mobile Robot. IEEE Trans. Control Syst. Technol..

[10] Yang H., Fan X., Shi P., Hua C. (2015). Nonlinear Control for Tracking and Obstacle Avoidance of a Wheeled Mobile Robot With Nonholonomic Constraint. IEEE Trans. Control Syst. Technol..

[11] Tzafestas S.G. (2018). Mobile Robot Control and Navigation: A Global Overview. J. Intell. Robot. Syst..

[12] Yue M., Wang L., Ma T. (2017). Neural network based terminal sliding mode control for WMRs affected by an augmented ground friction with slippage effect. IEEE/CAA J. Autom. Sin..

[13] Ren C., Li X., Yang X., Ma S. (2019). Extended State Observer-Based Sliding Mode Control of an Omnidirectional Mobile Robot With Friction Compensation. IEEE Trans. Ind. Electron..

[14] Ding X., Wang Z., Zhang L. (2021). Hybrid Control-Based Acceleration Slip Regulation for Four-Wheel-Independent-Actuated Electric Vehicles. IEEE Trans. Transp. Electrif..

[15] Singh P., Nandanwar A., Behera L., Verma N.K., Nahavandi S. (2021). Uncertainty Compensator and Fault Estimator-Based Exponential Supertwisting Sliding-Mode Controller for a Mobile Robot. IEEE Trans. Cybern..

[16] Xie Y., Zhang X., Zheng S., Ahn C.K., Wang S. (2021). Asynchronous H∞ Continuous Stabilization of Mode-Dependent Switched Mobile Robot. IEEE Trans. Syst. Man Cybern. Syst..

[17] Li J., Wang J., Peng H., Hu Y., Su H. (2022). Fuzzy-Torque Approximation-Enhanced Sliding Mode Control for Lateral Stability of Mobile Robot. IEEE Trans. Syst. Man Cybern. Syst..

[18] Xiong R., Li L., Zhang C., Ma K., Yi X., Zeng H. (2022). Path Tracking of a Four-Wheel Independently Driven Skid Steer Robotic Vehicle Through a Cascaded NTSM-PID Control Method. IEEE Trans. Instrum. Meas..

[19] Ailon A., Zohar I. Controllers for trajectory tracking and string-like formation in wheeled mobile robots with bounded inputs. Proceedings of the Melecon 2010—2010 15th IEEE Mediterranean Electrotechnical Conference.

[20] Yang H., Fan X., Xia Y., Hua C. (2015). Robust tracking control for wheeled mobile robot based on extended state observer. Adv. Robot..

[21] Dian S., Han J., Guo R., Li S., Zhao T., Hu Y., Wu Q. (2019). Double Closed-Loop General Type-2 Fuzzy Sliding Model Control for Trajectory Tracking of Wheeled Mobile Robots. Int. J. Fuzzy Syst..

[22] Chen C.-Y., Li T.-H.S., Yeh Y.-C., Chang C.-C. (2009). Design and implementation of an adaptive sliding-mode dynamic controller for wheeled mobile robots. Mechatronics.

[23] Yang X., Wei P., Zhang Y., Liu X., Yang L. (2019). Disturbance Observer Based on Biologically Inspired Integral Sliding Mode Control for Trajectory Tracking of Mobile Robots. IEEE Access.

[24] Taghavifar H., Rakheja S. (2020). A Novel Terramechanics-Based Path-Tracking Control of Terrain-Based Wheeled Robot Vehicle With Matched-Mismatched Uncertainties. IEEE Trans. Veh. Technol..

[25] Chiu C.-S. (2012). Derivative and integral terminal sliding mode control for a class of MIMO nonlinear systems. Automatica.

[26] Peng S., Shi W. (2017). Adaptive Fuzzy Integral Terminal Sliding Mode Control of a Nonholonomic Wheeled Mobile Robot. Math. Probl. Eng..

[27] Peng S., Shi W. (2018). Adaptive Fuzzy Output Feedback Control of a Nonholonomic Wheeled Mobile Robot. IEEE Access.

[28] Ding F., Huang J., Wang Y., Zhang J., He S. (2017). Sliding mode control with an extended disturbance observer for a class of underactuated system in cascaded form. Nonlinear Dyn..

[29] Huang J., Ri S., Fukuda T., Wang Y. (2019). A Disturbance Observer Based Sliding Mode Control for a Class of Underactuated Robotic System With Mismatched Uncertainties. IEEE Trans. Autom. Control.

[30] Liu K., Ji H., Zhang Y. (2019). Extended state observer based adaptive sliding mode tracking control of wheeled mobile robot with input saturation and uncertainties. Proc. Inst. Mech. Eng. Part C J. Mech. Eng. Sci..

[31] Jeong S., Chwa D. (2021). Sliding-Mode-Disturbance-Observer-Based Robust Tracking Control for Omnidirectional Mobile Robots With Kinematic and Dynamic Uncertainties. IEEE/ASME Trans. Mechatron..

[32] Matraji I., Al-Durra A., Haryono A., Al-Wahedi K., Abou-Khousa M. (2018). Trajectory tracking control of Skid-Steered Mobile Robot based on adaptive Second Order Sliding Mode Control. Control Eng. Pract..

[33] Zhai J.-Y., Song Z.-B. (2018). Adaptive sliding mode trajectory tracking control for wheeled mobile robots. Int. J. Control.

[34] Ye T., Luo Z., Wang G. (2020). Adaptive sliding mode control of robot based on fuzzy neural network. J. Ambient. Intell. Humaniz. Comput..

[35] Szczepanski R., Tarczewski T., Grzesiak L.M. (2019). Adaptive state feedback speed controller for PMSM based on Artificial Bee Colony algorithm. Appl. Soft Comput..

